# Multifunctional Coatings of Titanium Implants Toward Promoting Osseointegration and Preventing Infection: Recent Developments

**DOI:** 10.3389/fbioe.2021.783816

**Published:** 2021-12-07

**Authors:** Xiaoxuan Lu, Zichen Wu, Kehui Xu, Xiaowei Wang, Shuang Wang, Hua Qiu, Xiangyang Li, Jialong Chen

**Affiliations:** Key Laboratory of Oral Diseases Research of Anhui Province, Stomatologic Hospital and College, Anhui Medical University, Hefei, China

**Keywords:** orthopedic titanium implants, titanium implants, osseointegration, anti-infection, functional coatings

## Abstract

Titanium and its alloys are dominant material for orthopedic/dental implants due to their stable chemical properties and good biocompatibility. However, aseptic loosening and peri-implant infection remain problems that may lead to implant removal eventually. The ideal orthopedic implant should possess both osteogenic and antibacterial properties and do proper assistance to *in situ* inflammatory cells for anti-microbe and tissue repair. Recent advances in surface modification have provided various strategies to procure the harmonious relationship between implant and its microenvironment. In this review, we provide an overview of the latest strategies to endow titanium implants with bio-function and anti-infection properties. We state the methods they use to preparing these efficient surfaces and offer further insight into the interaction between these devices and the local biological environment. Finally, we discuss the unmet needs and current challenges in the development of ideal materials for bone implantation.

## Introduction

With the aging of the population, the incidence of orthopedic diseases has increased, and the use of orthopedic implants has increased rapidly. Titanium and its alloys, exhibiting stable chemical properties and excellent biocompatibility, are often used as materials for orthopedic implants. However, approximately 10% of implants need to be renovated due to their undesirable properties (Kurtz et al., 2005), among which aseptic loosening and infection of the implant are the main reasons for the failure. A study shows that in 337,597 procedures of knee revision, the infectious factors accounted for approximately 20.3% of implant failures, while aseptic loosening accounted for 20.4% (Delanois et al., 2017).

Aseptic loosening is mainly caused by the tiny gaps of prosthesisbone interface (Goodman, 1994; Sundfeldt et al., 2006). In effect of the gravity and pressure, the tiny gap increased and the wear particles will accumulate at the interface and hinder the direct contact between the implant and bone (Schmalzried et al., 1992; Sundfeldt et al., 2006). Accompanied with local inflammation and cascade reactions activated by immune cells, the osteoclast was enriched and bone resorption (Amstutz et al., 1992) occurred, which lead to implant loosening (Valstar et al., 2002). Besides, this process may also cause the transfer of bacteria from oral cavity to implant through the loosened gaps (Zhang, 2014). With the formation of bacteria colonization and biofilm, the undesirable milieu emerged, and even the local immune environment was destroyed, thus making antibiotics invalid to bacteria in the biofilm (Kaestner, 2016). Ultimately, implant failure would happen.

Therefore, aseptic loosening and infection are key risks to orthopedic implants. Long-term stability of orthopedic or orthodontic implants lies on the excellent osseointegration and antibacterial performance, which would do favor to establish a stable microenvironment and in turn restrain biofilm formation. Furthermore, the immune system, which is the first responder to the external microorganism and device, cannot be ignored. Xue et al. reviewed the surface modification of Ti and its alloys with the deep sight on physical and chemical techniques, including plasma spray, chemical vapor deposition, and microarc oxidation (MAO) (Xue et al., 2020). While taking more attention to the service environment of implants, this article mainly focuses on the latest report about how materials interact with the local biological milieu, especially with the microbe, osteocyte, and immune cells.

## Prevention of Aseptic Implant Loosening

**FIGURE 1:**
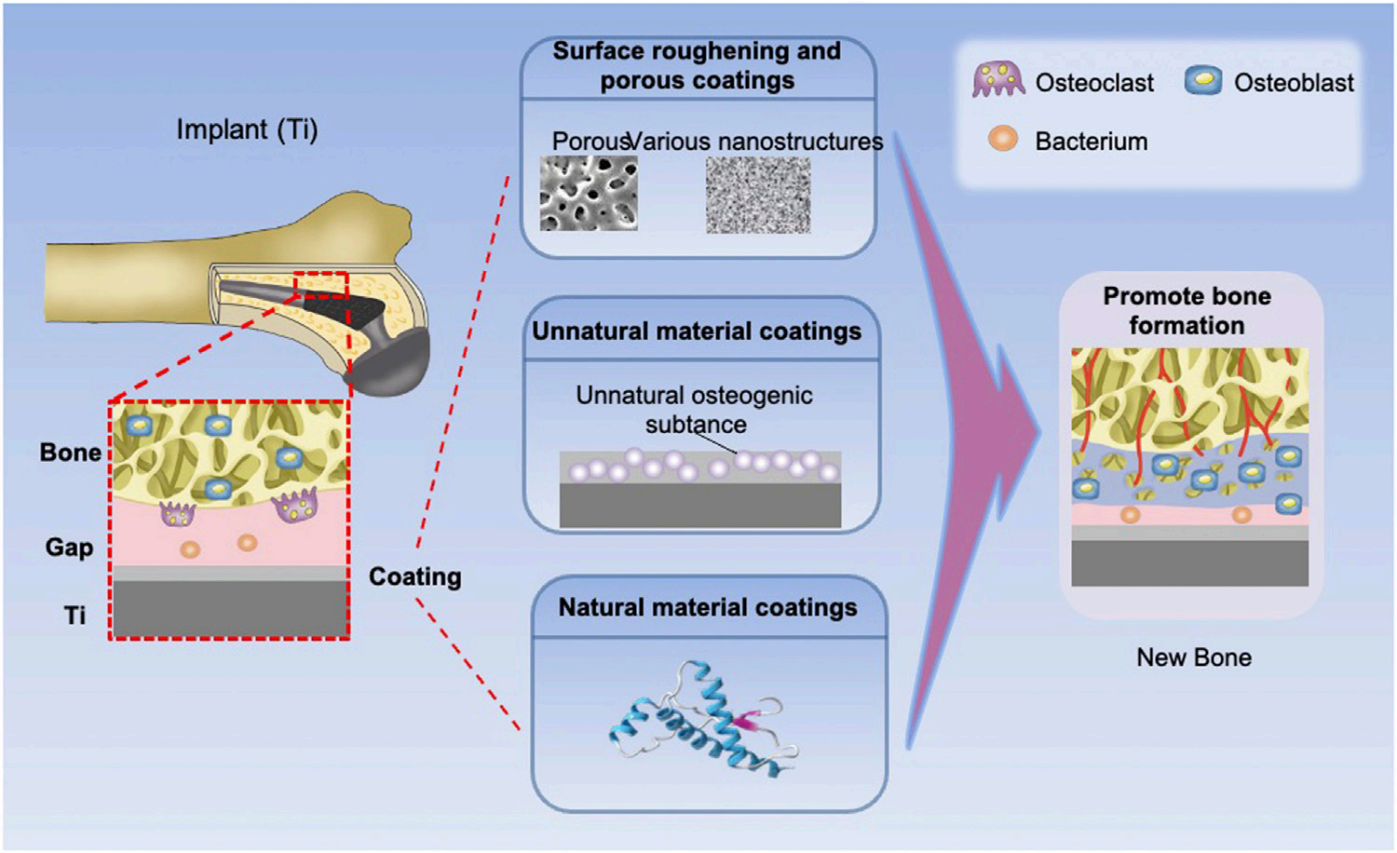
Types and effects of osteogenic coatings on titanium implants. The elastic coating can fill the interface gap by expansion. The implants with surface morphology coatings, inactive coatings, and bioactive coatings can promote new bone formation.

Osseointegration is a dynamic process during which the cells around implant secrete various cytokines to promote osteoblast recruitment and induce osteogenic differentiation to achieve bone formation (Sims and Martin, 2014). The osseointegration rate determines the bone remodeling and the sealing speed of the interface between bone and implant. A primary strategy is to construct a functional coating with osteoconduction or osteoinduction to promote osseointegration. In this section, three main categories of coatings for promoting osseointegration are introduced (Figure 1; Table 1).

**TABLE 1:** Recent development of osteogenic coatings on titanium implants

Category	Main methods or substances	Species of cell	Osseointegration function	References
Surface morphology coatings	Microarc oxidation (MAO)	MC3T3-E1 pre-osteoblasts	↑Adhesion, proliferation, differentiation, and mineralization	Zhao et al. (2020a)
	Dealloying	EA.hy926	↑Early osseointegration *in vivo*	Bai et al. (2017), Bai et al. (2018a), Bai et al. (2018b)
	3D printing	RAW 264.7	↑Biological activity of Ti implants	Wang et al. (2021a)
	Selective laser melting	BMSCs	↓Inflammatory response of macrophages	Ma et al. (2018)
	Porous/nanoporous	Human 1.19 fetal osteoblast-like (hFOB)	↑Osteoimmunomodulation to facilitate osteo/angiogenesis	Yu et al. (2020a), MacBarb et al. (2017)
			↑Angiogenesis, osteogenesis, and osteointegration	Song et al. (2019)
	Electrochemical anodizationNanotubes	MC3T3-E1	↑Proliferation of the osteoblast cells	Yao et al. (2020)
		HMSCs	↑Cell differentiation	Hu et al. (2020)
		BMSCsMice bone marrow-derived macrophages (BMDMs)	Nanotubes with different nanostructure of 80–100 nm were more likely to induce macrophages to the M1 phenotype, while nanotubes with smaller diameters of 30 nm were prone to induce macrophages to the M2 phenotype	Wang et al. (2018b)
	Alkali treatment nanonetwork structures (TNS)	rBMMSCs MG63 cell	↑Early cell adhesion and proliferation	Zeng et al. (2019)
			↑Osteogenesis and osseointegration	Yin et al. (2019)
			↑Osteogenic gene expression and mineralization	Huang et al. (2019b)
			↑Osteoprotegerin secretion	
	Vacuum diffusion bonding of titanium meshes	rBMMSCs	↑Cell adhesion, proliferation, and differentiation	Chang et al. (2016)
	Porous scaffolds with a variety of pore size and porosity			
	Template-assisted plasma spraying technique	BMSCs RAW 264.7	↑Polarization of macrophage to anti-inflammatory type M2	Zhang et al. (2018c)
	Patterned surface		↑Mineralization	
			↑Osteogenesis	
			↑Osteoimmunomodulatory properties	
	Microsecond laser direct writing and femtosecond laser-induced methods	MC3T3-E1	↑Cell adherence, alignment, and proliferation ↑Osteointegration	Li et al. (2020a)
	Micro-hexagons and nano-ripples			
	Acid etching and anodization	BMSCs	Nanotubes with different nanostructure of 80–100 nm were more likely to induce macrophages to the M1 phenotype, while nanotubes with smaller diameters of 30 nm were prone to induce macrophages to the M2 phenotype	Wang et al. (2018b)
	Nanotubes with different nanostructure	Mice bone marrow-derived macrophages (BMDMs)		
Inactive coatings	Mg/Ag/HA	Primary human osteoblasts	↑Adhesion, proliferation, and differentiation	Huang et al. (2018b)
		Human osteoblastic SaOS-2 cells	↑Osteogenic	Yao et al. (2021)
		Murine C3H10T1/2 cells		
				Ke et al. (2019)
	MPs-Sr	MC3T3-E1	↑Attachment and spreading of preosteoblast MC3T3-E1 cells	Zhang et al., 2018a
			↑Collagen secretion and matrix mineralization levels of cells	
			↑Osteogenic properties	
	Cu	RAW 264.7 Human osteoblastic SaOS-2 cells	↑Polarize to M1 phenotype	Huang et al. (2018a)
			↑Pro-inflammatory cytokines	
			↑Macrophage-mediated osteogenesis	
	Reduced graphene oxide	hMSCs	↑Proliferation and osteogenic differentiation of hMSCs	Jin et al. (2015), Kang et al. (2021)
			↑Cell adhesion and protein adsorption	
			↑Matrix mineralization	
	Glutamic acid/dopamine methacrylamide	MC3T3-E1	↑Calcium phosphate (CaP) formation with a Ca/P ratio close to that of natural hydroxyapatite	Long et al. (2020)
			↑Mineral deposition	
			↑Adhesion and proliferation of osteoblasts	
Bioactive coatings	BMSCs/BMP-2	rBMSCs	↑Biocompatibility and osteogenic differentiation ability	Bai et al. (2020)
			↑Osseointegration efficacy	
	Physisorption of fibronectin	MG63 cell	↑Osteoblast compatibility	Chen et al. (2020a)
	ADSC-EV		↑Osteoinductive activity	
	CaP-BMP2	Murine bone marrow mesenchymal stem cell line D1	↑Osteogenesis and angiogenesis	Teng et al. (2019)
			↑Bone formation	
	BMP-9	MC3T3-E1	↑Osteoblast proliferation and differentiation	Souza et al. (2018)
		BMSCs	↑Osseointegration	Zhu et al. (2021)
	Nerve growth factor (NGF)	—	↑Osseointegration	Lee et al. (2015)
			↑Nerve regeneration of peri-implant tissues	Ye et al. (2021)
	GO/IL-4	BMSCs	↑Biocompatibility	Li et al. (2020b)
		RAW 264.7	↑Macrophages polarization to the M2 phenotype *in vitro*	
			↑Proliferation, migration, and osteogenic differentiation of BMSCs	
	OGP-NAC	RAW 264.7	↓Important transcription factors for osteoclastogenesis	Liu et al. (2019)
			↑Osteoblast proliferation and differentiation	

### Surface Morphology Coatings

Osseointegration rate of orthopedic/dental implants may be attributed to their biochemical property and surface roughness, among which constructing a rough surface on Ti implant that does favor to bone anchoring and provides primary stability is a relatively mature strategy. In clinical trials, the roughened surface could increase the amount of translocated bone particles and provide greater contact area, both leading to outstanding osteogenic responses (Bencharit et al., 2014; Lou et al., 2015). Roughened surfaces were fabricated by various methods. In this section, we classify rough coatings into three categories from the different surface morphologies: porous, various nanostructures, and biomimetic coatings.

#### Porous Coatings

Porous coatings are one of the most common modifications of titanium surface morphology with a facilitative effect on osseointegration. Novel methods of preparing porous coatings include MAO, dealloying, and 3D printing. MAO can form closely bound porous structures on the titanium surface to promote osteoblast adhesion and proliferation (Zhao et al., 2020a). It was originally used for constructing in-grow oxidation ceramic layers on nonferrous metals (Van et al., 1977). By means of the approved mechanical and anticorrosion properties (Vovna et al., 1998; Gnedenkov et al., 2000), MAO was considered an outstanding method for implant modification to improve their bioactivity (Zhou et al., 2014). However, the sub-microscale pores on the MAO coating limits the in-growth of mature bone tissue (Liu et al., 2011). Some researchers proposed a hybrid treatment of MAO followed with hydrothermal synthesis to form a uniform apatite crystal layer on titanium surface, which promoted the interlocking between implant and bone (Huang et al., 2004). After that, Zhou et al. fabricated double-level porous coatings on Ti plates by three-step MAO (Zhou et al., 2015). Compared with the MAO coating treated with post-heat treatment, this strategy possessed better osseointegration and mineralization ability owing to the OH functional group, which promotes the synostosis between new bone and the implant (Bai et al., 2017). Furthermore, grafting metal ions and compounds is also a common method to improve the osteogenic ability of MAO coating. Some researchers used MAO to prepare manganesetitanium dioxide (Mn-TiO_2_) microporous coatings on titanium surface, which showed good biocompatibility and osteogenic property (Zhao et al., 2020a). Meanwhile, the Mn^2+^ release from the coating promotes surface mineralization, which would also favor osteogenesis. Bai et al. prepared a microporous TiO_2_ coating with MAO and modified with hydroxyapatite (HA) nanoparticles (Bai et al., 2018a). The microarc oxidation coating annealed at 650°C (MAO-650) had excellent physical and chemical properties. The resultant porous coating significantly promoted the proliferation and differentiation of osteoblasts and additionally inhibited the inflammatory reaction, realizing the dual function of immune regulation and bone formation. However, producing the homogeneous and crack-free coatings by MAO is still challenging (Yu et al., 2020b). Dealloying creates porous structures on alloy through a selective corrosion, preferentially dissolving the active alloying elements to form mono or hierarchical porous structures (Song et al., 2018). Wang et al. formed nanoporous structures on Ti-3Zr-2Sn-3Mo-25Nb (TLM) Ti alloy surfaces via dealloying (Wang et al., 2021a). Compared with hierarchical micro/nanoporous surface, mono structure has a higher hydrophilic and protein adsorption capacity, which is more conducive to the early adhesion of osteoblasts.

3D printing is the construction of a three-dimensional object from a digital 3D model, which can accurately control the surface morphology and integrate calcium phosphate, cytokines, and other osteogenic substances on the implant surface. Ma et al. prepared porous titanium alloy scaffolds customized to bone defects by 3D printing (Ma et al., 2018). The HA nanocrystals and collagen fibers induced by the 3D printed porous structure significantly promoted vascularized bone tissue formation in rabbit radius defect models, and the new bone formed a stable combination with the implant. However, in the process of 3D printing, the unmelted or partially melted particles will inevitably attach to the titanium surface to form rigid residues, which can cause chronic inflammation and osteolysis (Zhao et al., 2016). Therefore, removing residual powders on the substrate of 3D printing needs to obtain better biocompatibility. For example, Yu et al. fabricated micro/nanostructures on the surface of 3D-printed Ti-6Al-4V via acid etching and hydrothermal treatment, which played a positive role on promoting the cell proliferation and adhesion (Yu et al., 2020a). In addition, some researchers used a porous titanium scaffold printed by selective laser melting (SLM), which is similar to 3D printing, to modulate the surface topology of the scaffold and then improve the osseointegration (Song et al., 2019).

#### Coatings of Various Nanostructures

Coatings with different nanostructures, including nanoparticles, nanorods, and nanotubes, were prepared on the titanium surface by techniques such as alkali heat treatment and electrochemical anodization (EA), improving the surface roughness and promoting osseointegration. Some researchers found that the TiO_2_ nanotubes (TNTs) could increase the adsorption of major proteins involved in osteoblast adhesion, thus enhancing osteogenesis (Grimes and Mor, 2009). Due to the larger surface area, TNTs were used in various osteogenic materials. EA is a surface modification approach that applied an external voltage to the metal plate in the electrolyte to grow a specific structure orderly. Yao et al. fabricated anodic oxide nanotubes and deposited calcium phosphate (CaP) on the surface of Ti and Ti-6Al-4V (Yao et al., 2020). The cell experiment showed that the nanotubes with a higher HA content were suitable for cell adhesion. It should be noted that the fluoride ions and internal stress would cause weak adhesion between TNTs and Ti substrates (Zhang et al., 2015; Cao et al., 2018). Hu et al. performed grain refinement in Ti substrate by high-pressure torsion processing, improving the adhesion between nanotubes and Ti. This strategy also increased the elastic modulus of TNTs and further improved osseointegration (Hu et al., 2020). Moreover, TNTs have also been used as carrier for drug release in recent years because of their simple preparation, controllable size, and high loading capacity (Wang et al., 2017a).

The alkali treatment can produce a homogeneous nanonetwork structure (TNS) on the titanium surface. The study by Zeng et al. showed that this nanostructure enhanced the wettability of Ti to blood and binding to fibrin, which facilitated cell attachment and tissue healing (Zeng et al., 2019). The significant increase in the amount of new bone around the implant also exhibited a greater ability to promote osseointegration. To further improve the adhesion of the surface, Yin et al. coated mussel adhesive protein (MAP) on the TNS, resulting in promotion of new bone growth (Yin et al., 2019). This novel structure has great potential for clinical applications in the dental and orthopedic fields.

Some researchers compared the biological characteristics of different nanostructure coatings. By controlling the duration of the steam-hydrothermal treatment, the researchers prepared HA nanoparticles and nanorods in different sizes on the surface of the MAO micropores of titanium (Bai et al., 2018b). By contrast, the nanoparticles were more conducive to osteogenesis due to the closer resemblance to the structure of natural bone and their larger specific surface area. Huang et al. prepared nanowire-like and nanometal-like topography on the titanium surface using different concentrations of NaOH, both of which showed good biocompatibility compared with untreated titanium, with Ti-5 showing the best osteogenic differentiation ability (Huang et al., 2019b).

#### Other Morphology Coatings

Recently, some researchers have found that surfaces with well-defined patterns exhibit higher levels of cell adhesion, proliferation, and differentiation (Chang et al., 2016). Zhang et al. fabricated patterned titanium coatings using a template-assisted plasma spraying technique (Zhang et al., 2018d). The surface was more conducive to the polarization of macrophage to anti-inflammatory type M2 and induced osteoblasts to exhibit more mineralization nodules. Inspired by the skin structures of the tree frog toe pads and the adhesion of the corrugated ridges on the scales of Morpho butterfly wings, Li et al. generated micro-hexagons and nano-ripples on titanium surface by microsecond laser (Li et al., 2020a). This structure provided microscale space for cell behaviors, enhancing mechanical interlocking at the boneimplant interface. Similarly, some researchers have designed biomimetic coatings inspired by bone structure. MacBarb et al. compared the response of osteoblasts on the 3D-printed trabecular-like titanium implant surface, the titanium plasma spray-coated surface with and without nanocrystalline HA coating (MacBarb et al., 2017). The results showed that 3D-printed trabecular-like surface could promote the earlier proliferation of osteoblasts and higher calcium production than other surfaces, holding promise for improving the osseointegration of orthopedic implants.

#### Unnatural Material Coatings

Since the composition of HA is similar to bone, it is widely used in bone tissue engineering (LeGeros, 2002). However, due to the mismatch in coefficients of thermal expansion between metal and HA (Sun et al., 2001), there are potential concerns about the low strength of the bond between implant and coating. Ke et al. prepared gradient HA coatings on a titanium surface by Laser Engineered Net Shaping (LENS) and plasma spray deposition technology (Ke et al., 2019). The gradient coating solved the problem of weak interfacial bond, prevented the diffusion of metal ions effectively, and improved the surface osteoconductivity. Besides HA, various metal ions (Ca^2+^, Sr^2+^, Mg^2+^) have been demonstrated to possess the property to promote osseointegration (Xu et al., 2020). One example of these coatings is a biomimetic mesoporous coating doped with strontium (MPs-Sr), proposed by Zhang et al. (2018a). The mesoporous structure and the doped Sr significantly upregulated the attachment and spreading of preosteoblasts, indicating their ability to promote osseointegration and new bone formation. Huang et al. doped Mg elements with osteogenic ability into micro/nanoporous coatings by MAO and hydrothermal treatment to form MgO nanorods, which were later heat treated to convert the nanorods into nanoparticles, further improving the biological properties of the coatings (Huang et al., 2018b). Others reported the nanostructured Mg(OH)_2_ films on Ti surface fabricated by hydrothermal treatment (Yao et al., 2021). The animal experimental results and genome expression analysis indicated that the coating with Mg ion release could activate BMP-4-related signaling pathways, thus promoting bone formation and regeneration. At the same time, the alkaline environment has also been shown to favor the deposition of HA, leading to further osteogenesis, which has been proved by various studies (Chen et al., 2020b; Zhao et al., 2020b). Furthermore, researchers prepared the Cu-containing surface to induce macrophages to polarize to M1 phenotype, which could release appropriate pro-inflammatory cytokines to form an inflammatory microenvironment, thereby promoting osteogenic differentiation of MSCs (Huang et al., 2018a).

Besides, non-metallic substances have also been explored for promoting osseointegration. Graphene is a novel nanomaterial with excellent mechanical properties. It is increasingly used in the biomedical field owning to their excellent electronic, optical, mechanical, and chemical properties. Moreover, graphene derivatives such as reduced graphene oxide (rGO) can enhance protein adsorption and cellcell interactions, and have extraordinary osteoinductive ability (Jin et al., 2015). Kang et al. used meniscus-dragging deposition technology to coat rGO on the titanium surface to fabricate an rGO-Ti substrate (Kang et al., 2021). *In vitro* experimental results showed that the coating enhanced cell proliferation and significantly promoted matrix mineralization. Inspired by the physiological function of bone sialoprotein during osteogenesis, Long et al. synthesized a polymer from glutamic acid and dopamine methacrylamide via reversible addition-fragmentation chain transfer polymerization and immobilized it on a titanium base by means of catechol pendants on the polymer chain (Long et al., 2020). The coating could induce calcium phosphate (CaP) formation with a Ca/P ratio close to that of natural hydroxyapatite, thus effectively promoting mineral deposition. In addition, the coating could promote the adhesion and proliferation of osteoblasts.

#### Natural Material Coatings

Cellular active substances such as extracellular matrix proteins, growth factors, and chemokines are hotspots of research for osseointegration, and their application on the surface of orthopedic prostheses can effectively improve the biocompatibility of the surface and promote osseointegration. These bioactive coatings could be classified into two parts: 1) directly loaded with osteogenic factors; 2) loaded with immunomodulatory factors. In this part, we will introduce the new progress of bioactive coating in recent years and discuss their application in detail.

#### Coatings Loaded with Osteogenic Factors

Stem cell encapsulation and extracellular vesicle loading that endow surfaces with bioactive property for rapid osteogenesis are two main strategies in constructing bioactive surfaces. Mesenchymal stem cells (MSCs) have been widely used in research and clinic because of their self-renewal potential and multilineage differentiation (Mushahary et al., 2018). Bone marrow is the prevailing source of MSCs and bone marrow mesenchymal stem cells (BMSCs) have a potential to differentiate into osteoblasts (Pittenger et al., 1999). Bai et al. encapsulated BMSCs, bone morphogenetic protein-2 (BMP2), and other bioactive substances in hydrogels and formed a three-dimensional inorganicorganic supramolecular bioactive interface on a porous titanium alloy scaffold (Bai et al., 2020). Osteogenic differentiation of BMSCs was induced by the continuous release of BMP2. With the degradation of the hydrogel, bone tissue grew into the pores of the scaffold to achieve good bone integration. However, since the harvesting procedure with general anesthesia limits the supply of BMSCs (Li et al., 2016), adipose tissue stem cells (ADSCs), another candidate for bone engineering that is easy be obtained, are attracting more attention (Kolaparthy et al., 2015). The main way to exert the benefit of MSC is to secrete extracellular vesicles (EVs) to promote tissue repair and regeneration (Vizoso et al., 2017; Mushahary et al., 2018). On one hand, MSC-EVs can carry and transfer various cargo such as regulatory miRNAs, growth factors, and cytokines. On the other hand, the membrane of EVs contains bioactive signaling molecules to obtain protection (Malda et al., 2016; Tsiapalis and O’Driscoll, 2020). Based on physisorption of fibronectin, Chen et al. immobilized adipose-derived stem cell-derived extracellular vesicles (ADSC-EVs) onto the titanium surface, which enhanced osteoblast compatibility and osteoinduction activity (Chen et al., 2020a).

Osteogenesis-related molecular loading is another strategy for constructing bioactive surface. Growth factors and cytokines could promote osteogenesis by upregulating the level of osteogenic differentiation-related genes or activating osteogenic-related signal pathways. Bone morphogenetic proteins (BMPs) are the widely used cytokines to confer osteoinductivity (Carson and Bostrom, 2007). However, a burst release will decrease the osteogenic effect (Haidar et al., 2009a; Haidar et al., 2009b). Teng et al. prepared a porous structure on the titanium surface by 3D printing and MAO to endow the coating with osteogenic property, calcium, phosphate, and BMP-2 that was grafted onto the surface (Teng et al., 2019). The results showed that the continuous BMP-2 release sustained for more than 35 days, and enhanced the osseointegration between the implant and surrounding bones. Compared with BMP-2, BMP-9 has higher osteoinductive differentiation ability (Kang et al., 2004; Souza et al., 2018). Zhu et al. implanted thermosensitive collagen and BMP-9 into porous titanium, which could release BMP-9 via temperature-controlled sustained release (Zhu et al., 2021). The thermosensitive collagen degraded slowly at 37°C and the released BMP-9 significantly promoted osteogenesis around the implant. Besides, sympathetic nerves are also widely distributed in bone tissue and can regulate bone formation. Nerve growth factor (NGF) has been shown to enhance the activity of osteoblasts and promote and mineralization after implantation (Lee et al., 2015). The NGFchondroitin sulfate/hydroxyapatite coating (NGF-CS/HA-coating) prepared by modified biomimetic method was placed in the mandible of beagles (Ye et al., 2021). The results showed that the coating significantly upregulated the level of osteogenesis differentiation-related genes in the mandible, promoting the differentiation of osteoblasts and nerve cells in the early bone binding and the bone healing around the implant.

#### Coatings Loaded with Immunomodulatory Factors

As a foreign body, the implant inevitably leads to a series of immune responses, which mainly arise from the macrophage activation that would reduce the service life of implants. In addition, as mentioned previously, the wear particles produced in the gap of implant and bones can also aggravate inflammatory reaction, and cause a dynamic imbalance between osteoblasts and osteoclasts, which eventually lead to bone resorption and implant loosening (Zhou et al., 2021). Therefore, tuning immunoreaction to keep an appropriate immune environment is beneficial to improve osseointegration and reduce loosening. There are many ways to regulate immune microenvironment. Previous studies have shown that different nanostructured Ti can induce different macrophage responses, which can affect the osseointegration (Wang et al., 2018b). Alternatively, some coatings loaded with immune factors can also regulate the immune response, such as interleukin 4 (IL-4) (Li et al., 2020b) and complement activating immunoglobulin (Harmankaya et al., 2012). Li et al. sprayed graphene oxide (GO) on Ti and loaded IL-4 to construct a GO/IL-4 coating for regulating macrophage-related inflammation (Li et al., 2020b). In the process of acute inflammation, type 1 macrophages (M1) can produce fibers to protect the host, while type 2 macrophages (M2) can inhibit the development of inflammation and promote the growth of osteogenic factors (Franz et al., 2011). The release of IL-4 from GO/IL-4 coating induced the macrophage polarization to the M2 phenotype, weakened the inflammatory response, and promoted osteogenesis. Another method for immune regulation is to regulate the balance between osteoblasts and osteoclasts. Liu et al. conjugated an osteogenic growth peptide (OGP) with N-acetylcysteine (NAC) to synthesize a multifunctional peptide OGP-NAC and then employed OGP-NAC to titanium (Liu et al., 2019). Such an OGP-NAC coating could inhibit the important transcription factors for osteoclastogenesis, such as MAPK, NF-κB, and NFAT c1, and promote osteoblast proliferation and differentiation.

#### Prevention of Implant Infection

Infections within 3 months are considered as early postoperative infection, while delayed (or subacute) infection occurs after 3–24 months and late infection more than 24 months later (Montanaro et al., 2011; Zimmerli, 2014). Early infection is usually caused by pathogens such as *Staphylococcus aureus* at the surgical site (Trampuz and Widmer, 2006). After operation, patients need systemic antibiotic treatment to prevent infections, but the rising antibiotic resistance of bacteria can make the existing antibiotics noneffective (Park et al., 2019). Also, the concentration of antibiotics in the focus site is insufficient, resulting in the rapid proliferation and secretion of extracellular polymers to form a biofilm after some pathogens gather and adhere to the implant surface (Gristina and Costerton, 1985). Exopolysaccharides of the biofilm can hinder and delay the penetration of antibiotics, and the quorum sensing of bacteria in the biofilm regulates the development of the biofilm to resist the host immune defense, thus making the biofilm a barrier to antibiotics (Kaestner, 2016). Therefore, the ideal antibacterial coating is supposed to remove or kill the pathogens once the primary contact occurs, thus preventing the formation of the biofilm. Orthopedic implants would maintain for a long time in the organism, so late infections would happen if no defensive measures were taken (Del Pozo and Patel, 2009). At this stage, the biofilm formed, resulting in poor antibiotic treatment (van de Belt et al., 2001), so it is necessary for the implant surface to provide long-term antibacterial properties.

**FIGURE 2:**
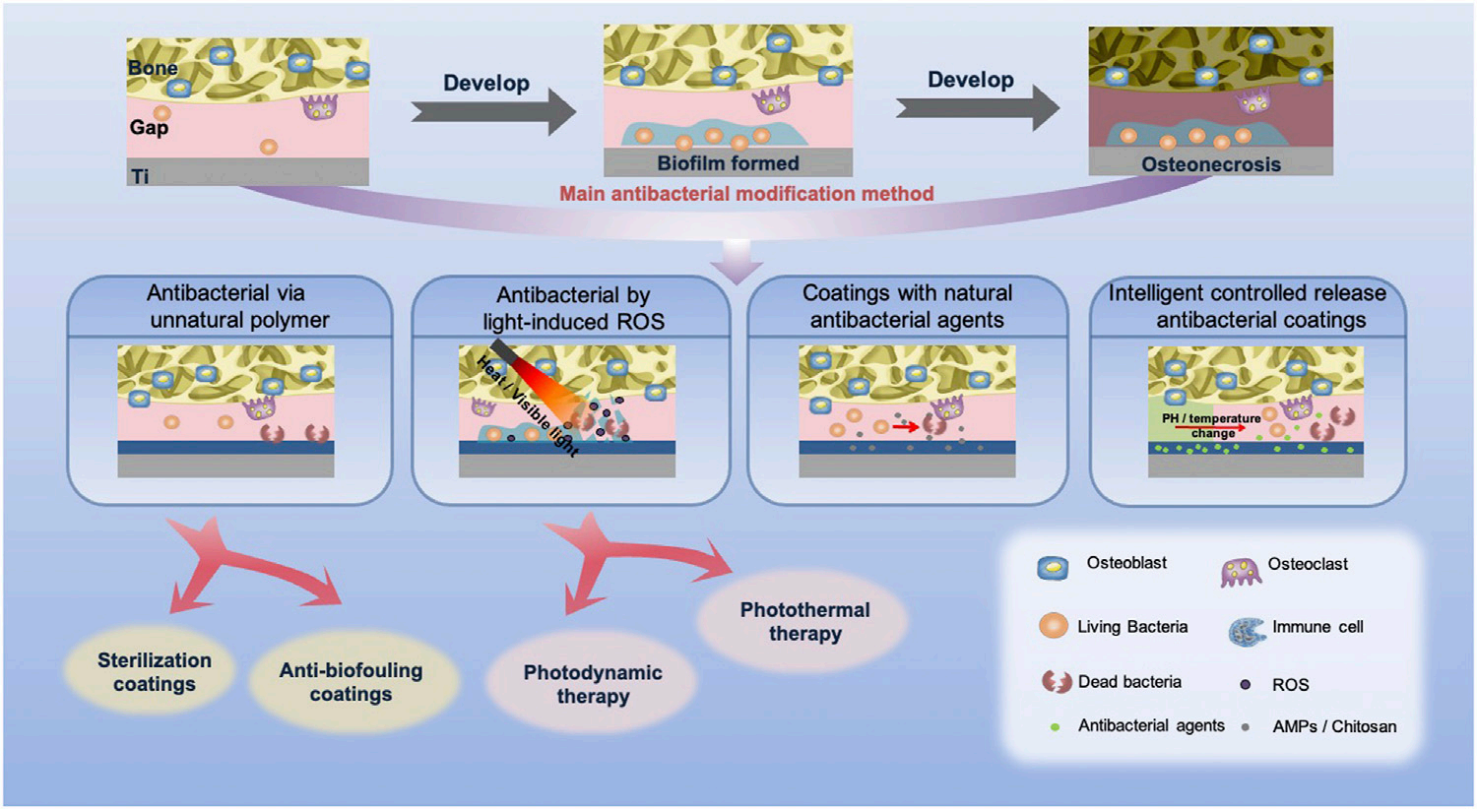
Types and effects of antibacterial coatings of titanium implants. After implantation, biofilm may be formed, leading to osteonecrosis in general. At present, to avoid infection, the coatings with inactive polymers, light-induced ROS and bioactive antibacterial agents, and the intelligent controlled release antibacterial coatings are the main antibacterial methods.

The key to preventing infection is to inhibit the adhesion of microorganisms, but long-term infection can still be a risk to the implant because of continuous pathogens. Therefore, the anti-infective implants should target early infection and long-lasting antibacterial agents. This section introduces preventive and treatment strategies of infections (Figure 2; Table 2).

**TABLE 2:** Recent development of antibacterial coatings on titanium implants

Categories	Main anti-infection agent	Mentioned synthesis method	Included bacteria species	Antibacterial effects	References
Metal ions	AgNPs	Physical vapor deposition (PVD)	*S. aureus*, *S. mutans*, *S. epidermidis*, *E. coli*, *P. aeruginosa*	↑Antibacterial effect	Lampé et al. (2019)
		Electron cyclotron resonance (ECR)			Chen et al. (2016)
		*In situ* dopamine reduction			Guo et al. (2020)
		Layer-by-layer assembly			Wang et al. (2021b)
		Electrodeposition			Ren et al. (2021)
		Thermochemical treatment			Rodríguez-Contreras et al. (2021)
	Zn	MAO	*S. aureus*	↑Antibacterial effect	Ye et al. (2020)
		Electrodeposition	*E. coli*		Shahmohammadi and Khazaei (2021)
		Hydrothermal method	*P. gingivalis*		Chen et al. (2021b)
	Cu	—	MRSA	↓Biofilm formation, virulence	Zhuang et al. (2021)
				↓Antibiotic resistance of MRSA	
	Ga	Hydrothermal method	*S. aureus*	Strong antibacterial ability	Li et al. (2021b)
			*E. coli*		
	AgNPs	Laser cladding	*S. aureus*	Long-term synergistic antibacterial activity of Zn^2+^ and Ag^+^	Zhang et al. (2018b)
	Zn^2+^		*E. coli*		
	AgNPs	Plasma electrolytic oxidation	MRSA	Synergistic antibacterial activity of Ag and Cu	van Hengel et al. (2020b)
	CuNPs				
Non-metallic antibacterial substances	Iodine	Anodization	*S. aureus*	↓Bacterial colonization	Shirai et al. (2011)
			*E. coli*		
	Chlorhexidine	Organosilane chemistry	*S. aureus*	↓Bacteria adhesion and growth	Wang et al. (2019)
Anti-fouling coatings	PEG	Simultaneous deposition; electrodeposition	*S. aureus*	↓Protein absorption	Guo et al. (2021)
			*E. coli*	↓Bacterial and platelet adhesion	Hoyos-Nogués et al. (2018)
			*S. sanguinis*	↓Biofilm formation	
	Zwitterionic copolymer	Free radical polymerization	*E. coli*	↓Protein adsorption, platelet adhesion	Huang et al. (2017)
		RAFT polymerization		↓Bacteria adhesion	
Light-induced ROS	Photodynamic-induced ROS	Plasma electrolytic oxidation	*S. sanguinis*, *A. naeslundii*	↑Antibacterial effect in light conditions	Nagay et al. (2019)
				↑Degradation efficiency of lipopolysaccharide	Wu et al. (2019)
	Photothermal-induced ROS	π-π stacking	*S. aureus*, MRSA	↑Bacteria killing	Yuan et al. (2019)
		Hydrothermal method		↓Biofilm formation	(Song et al., 2020)
		EDC-NHS chemistry			Su et al. (2020)
		Sulfur doping			
Bioactive antibacterial agent	AMP	Organosilane chemistry, click chemistry	*S. gordonii*, *S. aureus*	↑Antibiofilm activity	Acosta et al. (2020)
		Layer-by-layer assembly			Rodríguez López et al. (2019)
Intelligent controlled release antibacterial coating	Gentamicin	—	*S. aureus*	↓Bacteria growth and adhesion	Sang et al. (2021)
			*E. coli*	↑Bacteria killing in a slightly acidic environment	
	Glycerin	Anodization	*S. aureus*	↑Immunoregulatory antibacterial activities at 40°C	Li et al. (2021a)
			*E. coli*		

#### Antibacterial *via* Unnatural Polymers

Some metal ions and polymers are widely used as antimicrobial agents coating the implants. In this section, we divide these unnatural antimicrobial agents, such as metal ions, non-metallic ions, and other synthetic polymers, into sterilization coatings and anti-biofouling coatings to describe them in detail.

#### Sterilization Coating

##### Antibacterial Metal Ions

Clinically, antibiotics have been widely used in the treatment of implant infection, but its clinical effect is limited due to the drug resistance of bacteria and the narrow antibacterial spectrum of single antibiotics. Therefore, metal ions with broad-spectrum antimicrobial effects that do not lead to severe resistance are deemed potential antibiotic substitutes for implant surface modification, such as Ag^+^, Cu^2+^, and Zn^2+^ (Sundfeldt et al., 2006). Chen et al. integrated silver nanoparticles on the surface of titanium by *in situ* reduction of dopamine (Chen et al., 2016). The results showed that the surface had excellent antibacterial activity, but Ag also showed strong cytotoxicity. To reduce the cytotoxicity, Guo et al. prepared a poly-l-lysine (PLL)/sodium alginated (SA)/PLL self-assembled coating on the surface of Ti, then loaded nano-silver and induced mineralization in simulated body fluid (Guo et al., 2020). The antibacterial results showed the coating effectively inhibited the adhesion of bacteria. At the same time, PLL/SA/PLL coating can greatly improve the cytocompatibility and reduce the cytotoxicity. Another potential idea is to decrease the cell cytotoxicity *via* adjusting the concentration of antimicrobial agents. Wang et al. systematically determined the osteogenesis of dopamine silver-loaded coatings prepared at different pH (4, 7, 10) and different Ag^+^ concentrations (0.01, 0.1 mg/ml) (Wang et al., 2021b). The results showed that the pH 10/0.1 group had obvious osteogenesis in bacterial environment, which may be due to its strong antibacterial properties to kill the surrounding bacteria and then promote mineralization to achieve good osseointegration. Also, other researches incorporated zinc-doped coating on titanium surface (Ye et al., 2020). The amorphous coating with ZnO bond acts as the Zn^2+^ donor and generator of reactive oxygen species (ROS), which could trigger the *S. aureus* killing. In addition, although Zn^2+^ and ROS can cause osteoblast damage, the surface did not show noteworthy cytotoxicity.

Since the antibacterial and cytotoxicity of Ag^+^ are both dose dependent, many researches focus on doping adjuvant antibacterial substance. Zhang et al. deposited HA nano-powder mixed with Ag and ZnO on a titanium surface by laser cladding (Zhang et al., 2018c). The combined release of Ag^+^ and Zn^2+^ for more than 5 weeks showed a long-term effective antibacterial activity. Moreover, with the assistance of Zn^+^, the antibacterial efficiency was significantly increased and cytotoxicity of the Ag^+^ contained coating was reduced. To reduce the negative performance of Ag^+^, Xie et al. prepared a hybrid coating containing chitosan (CS), dopamine (PDA), HA, and nano-silver. The double chelation of PDA and CS achieved long-term release of silver and a continuous bacteriostatic effect. It is worth noting that this coating exhibits significantly osteogenic potential in both *in vitro* and *in vivo* tests (Xie et al., 2019). However, the antibacterial substances released by this kind of coating gradually decrease over time, and with the adhesion of proteins and host cells, the antibacterial property will decrease. Therefore, the long-term and stable antibacterial effect is still a challenge in the development of antimicrobial coatings.

##### Non-Metallic Antibacterial Substances

In addition to antimicrobial metal ions, some non-metallic compounds and biomolecules are also prominent candidates for fabricating antibacterial coatings, among which iodine is found to have a wide antibacterial spectrum and do not develop drug resistance (Tsuchiya et al., 2012). Shirai et al. described the novel use of povidone-iodine as the electrolyte to form the iodine coating on the Ti surface (Shirai et al., 2011). After implanting into the femora of rabbits, the implants significantly inhibited the formation of biofilm. Meanwhile, since iodine is an important component of the thyroid hormone, the coating has important biological safety. Since then, Kabata et al. treated 28 patients with iodine-supported implants (Kabata et al., 2015). None of the patients showed infection after 1-year follow-up, indicating that the coating could effectively prevent postoperative infections. Others used the same method to prepare iodine coatings and investigated temporal changes in iodine for implants using animal models for 1 year (Kato and Shirai, 2016). The results showed that the implants retained 30% of the iodine content after 1 year, which proved to have sufficient antibacterial properties. Similarly, chlorhexidine is a commonly used clinical antibacterial agent (Kim et al., 2019), which can be adsorbed on the surface of bacteria to destroy membrane permeability, thus sterilizing. Wang et al. covalently grafted chlorhexidine onto porous titanium through the coupling ability of aminosilane and glutaraldehyde (Wang et al., 2019). The results showed that chlorhexidine could inhibit the adhesion and proliferation of bacteria. What is more, in the cell-bacterial competition environment, this surface is of great help to osteoblast adhesion.

##### Anti-Biofouling Coatings

The anti-biofouling coatings can change the hydrophilicity of the surface, thus repelling the adhesion of bacteria and proteins. Polyethylene glycol (PEG) is the most common anti-biofouling material that is highly hydrophilic, which can constitute a wide exclusion volume that repels bacterial contamination (Tanaka et al., 2010). Guo et al. deposited both polyphenol tannic acid and PEG on the titanium surface, providing good inhibition of non-specific adsorption of proteins, adhesion of bacteria and platelets, and prevented biofilm formation (Guo et al., 2021). However, since its rejection is non-specific, the coating repels contamination while also inhibiting cell adhesion (Tanaka et al., 2010). The researchers introduced RGD sequence and antimicrobial peptides onto the PEG layer to obtain both cell adhesion and antimicrobial properties (Hoyos-Nogués et al., 2018).

It should be noted that PEG is easily oxidized in biological environment due to its poor stability (Guo et al., 2021). Zwitterionic copolymer is a hydration layer that can form a close bond on the surface of the material, and it is an ideal substitute for PEG (Xie et al., 2012). Huang et al. prepared a phosphonatezwitterionic block copolymer from sulfobetaine methacrylate and phosphonate/phosphonic methyl methacrylate, and immobilized it on titanium sheets (Huang et al., 2017). The results showed that this copolymer effectively inhibited protein adsorption, platelet adhesion, and bacterial adhesion, significantly improving the anti-biofouling ability of the titanium base. In addition, Salvagni et al. applied elastin-like recombinamer to modify the titanium base (Salvagni et al., 2014). The coating was capable of reducing serum-protein adsorption and was biologically active compared with the untreated titanium base, laying the groundwork for future medical applications. However, non-specific anti-biofouling still needs to be addressed.

### Antibacterial by Light-Induced ROS

Reactive oxygen species (ROS) can destroy the polysaccharides, which is the main component of the external surface of biofilm, thus representing bactericidal effect to drug-resistant bacteria (Konopka and Goslinski, 2007). Photosensitizers such as TiO_2_ and ICG can produce ROS when exposed to light. This section introduces the new progress in antimicrobial activity through light-induced ROS.

#### Photodynamic Therapy

Photodynamic therapy can destroy the biofilm by creating free radicals and then disrupting the biofilm (Konopka and Goslinski, 2007). Sharab et al. combined this therapy with physical methods and applied it to the combating with biofilm of *Streptococcus mutans*, showing that photodynamic therapy can weaken biofilms of different maturity levels (Sharab et al., 2020). Titanium dioxide (TiO_2_) is a stable photocatalyst, which can produce ROS to kill bacteria under ultraviolet (UV) irradiation (Carp, 2004), but UV is harmful to the human body. To solve this problem, Nagay et al. prepared nitrogen and bismuth doped TiO_2_ coatings by plasma electrolytic oxidation of titanium (PEO), which produces ROS to kill bacteria under visible light (Nagay et al., 2019). Others used PEO to prepare TiO_2_ nano-ceramic coating on titanium surface to kill bacteria and decompose organic residues under visible light irradiation, and effectively prevent the occurrence of peri-implant inflammation (Wu et al., 2019). However, since this strategy is light dependent, they are only fit for oral implants.

#### Photothermal Therapy

In recent years, photothermal therapy (PTT) based on near infrared radiation has become a hot spot in the field of nanomedicine because it is less traumatic than UV light, allows deep tissue penetration, and has high selectivity (Cheng et al., 2014). PTT can destroy bacterial integrity or biofilm structure through local warming (Hu et al., 2017; Wang et al., 2018a; Li et al., 2018; Qiao et al., 2019), but its unselective heating may adversely affect the surrounding tissue (Lei et al., 2016; Tan et al., 2018). Therefore, PTT has a higher application potential in combination with other antibacterial strategies. Zhang et al. combined indocyanine green (ICG) with mesoporous polydopamine (mPDA) to form a multi-functional coating on titanium surface, using mPDA to convert light energy into heat for microorganism killing, and the ICG can also produce ROS under light radiation to destroy the bacterial cell wall (Yuan et al., 2019). This strategy can be remotely controlled to eradicate the biofilm formed on the surface of implants *in vivo*, avoiding debridement surgery or invasive treatment, and does not cause side effect to surrounding tissue. Song et al. used dopamine and ferrocene (PDA-Fc) to modify TiO_2_ nanorods as an antibacterial coating on titanium surface (Song et al., 2020). Local high temperature was attained by photothermal transformation of PDA, and ROS were produced by PDA-Fc redox reactions, which achieve a synergistic and more efficient bactericidal performance.

Photodynamic TiO_2_ is not only a typical photosensitizer but also sound sensitizers, so ROS can be produced by ultrasound-triggered electron hole separation (Harada et al., 2011). Su et al. proposed a photoacoustic therapy, which produced oxygen defects on titanium implants by sulfur doping (Ti-S-TiO_2-x_), to endow the implants with good sonodynamic and photothermal properties (Su et al., 2020). The killing rate of *S. aureus* was as high as 99.995% after 15 min of exposure to near-infrared light and ultrasound. In addition, the implant showed good stability, and the structure and properties did not change after soaking in water for 6 months.

### Antibacterial *via* Natural Antibacterial Agents

Antimicrobial peptides (AMPs) are the most widely studied antibacterial agents in recent years, which are natural anti-infective agents. AMPs are general constituted by two parts: one part is composed of positively charged residues like arginine and lysine, which will do favor to the initial contact; another are hydrophobic residues that may penetrate into the cell to bind intracellular molecules, thus killing the microorganism (Hancock and Sahl, 2006). Shi et al. loaded AMP, Tet213, on titanium via layer-by-layer technique, which effectively inhibited the early *S. aureus* biofilm formation (Shi et al., 2015). Furthermore, this multilayer coating could release AMP continuously for up to a month. Since the function of the AMP is closely related to their conformation, the surface grafting based on covalent bond would inevitably reduce their antibacterial efficiency (Steckbeck et al., 2014). Therefore, mild methods were developed to prepare AMP-loaded coatings without reducing their activity. The antimicrobial peptide GL13K extracted from parotid secretory protein also showed potent antimicrobial activity. To improve the stability, the researchers replaced the lysine residues in GL13K with d-amino acids, generating an all-d-amino acid version of GL13K (D-GL13K), which reduced the solubility of the peptide (Hirt and Gorr, 2013). Based on this, Acosta et al. developed an extracellular matrix system based on elastin-like recombinant (ELR), an extracellular matrixinspired polymer, to attach the D-GL13K to a titanium surface (Acosta et al., 2020). On the one hand, ELR coating could reduce the adsorption of non-specific proteins (Salvagni et al., 2014), thereby giving the surface antifouling performance. On the other hand, D-GL3K has good bactericidal activity and can reduce the adhesion of bacteria and the formation of biofilm.

Similarly, chitosan, a natural polymer, is widely used in tissue engineering due to its good biocompatibility (Ordikhani and Simchi, 2014). Chitosan is a cationic macromolecule that can bind negatively to cell membrane of bacteria that exhibit antibacterial property (Raafat and Sahl, 2009), but the antibacterial activity is too weak that limits its use on implants. To construct the appropriate antibacterial coatings, other antibacterial agents like AMPs are used to develop an antibacterial composite (Raghavendra et al., 2017; Tabesh et al., 2019). López et al. use chitosan (CH) and hyaluronic acid (HA) polyelectrolyte multilayers (PEM) loaded with β-peptide (a kind of antimicrobial peptide), prepared by layer-by-layer electrostatic assembly technology (Rodríguez López et al., 2019). By the strong chemical cross-link of CH/hyaluronic acid thin films, a prolonged β-peptide retention about 8 weeks was obtained and significant inhibition of *S. aureus* biofilm was achieved. In addition, the multilayer film retained its antibacterial activity after five attacks by five separate bacterial over 18 days. Such a strategy that can maintain a long-term antibacterial property is critical for the viability of the Ti implants.

### Intelligent Controlled Release Antibacterial Coatings

As mentioned previously, despite the potent antibacterial activities, certain cytoxicities of antimicrobial agents remain a concern. Since the cytoxicity is dose dependent, the control release that can tune local concentration of the antimicrobial agents and prolong the effect time is a significant direction. At present, the control release behavior mainly relies on pH and temperature to realize the intelligent release of antibacterial agents (Li et al., 2021a; Sang et al., 2021). As mentioned earlier, antibiotics not only cause drug resistance of bacteria but their rapid release can also produce cytotoxicity. In this regard, some researchers prepared controlled release antibiotic coatings to selectively release drugs when the microenvironment was changed. Silk fibroin that can respond to slightly acidic environment is regarded as a new candidate for drug loading and release (Hassani Besheli et al., 2017). Sang et al. coated a layer of silk protein on the surface of Ti, and then loaded gentamicin (Sang et al., 2021) on it. Bacteriostatic ring tests *in vitro* and *in vivo* showed that the coating exhibits a stable release behavior of gentamicin, and the release rate of gentamicin in acidic environment was faster than that in alkaline environment, which achieved the intelligent release. Another mechanism for intelligent controlled release is to use the heating effect caused by infection. Li et al. layered a thermosensitive chitosan-glycerol-hydroxypropyl methyl hydrogel (CGHH) on TiO_2_ nanotubes (Li et al., 2021b). *In vitro* antibacterial test showed that CGHH had almost no antibacterial activity against *E. coli* and *S. aureus*. However, the results of subcutaneous infection animal models showed that under the high temperature caused by an infection *in vivo*, CGHH could release a large amount of glycerin, thus suggesting excellent antibacterial properties.

### Strategies of Both Preventing Aseptic Loosening and Infection

Promoting osseointegration along with the prevention of infections is the key factor of the orthopedic implants, but most of the current implant coatings exhibit only one of the properties. Since many antibacterial surfaces not only inhibit bacterial colonization but also do damage to mammalian cells, strategies to prepare host-friendly devices was the main direction of the orthopedic implants. In this review, several approaches that address the two properties at one surface were displayed, and we classify the coatings into four categories based on their characteristics (Table 3): 1) rough surface coating loaded with antibacterial substance; 2) osteogenic antibacterial polymer coatings; 3) photoantibacterial and osteogenic coatings; 4) multifunctional coatings with single material.

**TABLE 3:** Recent development of multifunctional coatings on titanium implants

Categories	Composition of surface coating	Associated coating strategy	Osteogenesis and antibacterial function	References
Osteogenic antibacterial polymer coatings	DMADDM/HA	Covalent immobilization by PDA	↑*In vivo* osteogenic differentiation and new bone formation	Zhou et al. (2020)
			↓Adherence and growth of pathogens	
	ALN/QPEI	Covalent immobilization by PGED brushes	↑Osteointegration and biomechanical properties	Sun et al. (2019)
			↓Bacterial infection	
	NCS/AMP	MAO	↑Osseointegration	Zhang et al. (2021)
		Hydrothermal treatment	↑Antimicrobial effect	
		Covalent immobilization		
	TNT/ZIF-67/OGP	EPD	↑Differentiation of MSCs	Tao et al. (2021)
			↓Inflammatory response	
			↑Antimicrobial effect	
Roughening/porous surface loaded with antibacterial substance	Mg/Zn-MOF74	Alkali-heat treatment	Leading to alkaline microenvironment	Shen et al. (2019)
		Thermal oxidation	↑New bone formation	
			↑*In vivo* antibacterial and anti-inflammatory properties	
	TNPC	Alkali-heat treatment	↑The formation of HA	Gao et al. (2020)
		Coordination bonds between catechol and TiO_2_	↓Pathogenic bacteria and biofilm formation	
	AgNP/ZnNP	SLM	↑Metabolic activity of pre-osteoblasts	van Hengel et al. (2020a)
		PEO	↑Antibacterial leaching activity against MRSA	
	PDA/LL-37/POPC	MAO	↑Cytocompatibility to MSCs and osteoblasts	He et al. (2018)
			↑Antibacterial activity against *S. aureus* and *E. coli*	
Photoantibacterial and osteogenic coatings	Collagen/PDA/MoS_2_-TiO_2_	MAO	↑Proliferation, adhesion, and spreading of osteoblasts	Han et al. (2021)
		Hydrothermal treatment	↓*In vivo* bacterial infection and *S. aureus* biofilm	
		Covalent immobilization		
	TiO2/UCN/Qr/LA	Hydrothermal in TMAOH	↑Angiogenesis and osseointegration	Zhang et al. (2022)
		Covalent immobilization	↑Antimicrobial effect	
		Electrostatical		
	TiO_2_/GDY	Electrostatic force	↑Bone tissue regeneration	Wang et al. (2020)
			↑Antimicrobial effect	
	RP/IR780/RGDC	Covalent immobilization by PDA	↑*In vivo* biostability and biocompatibility	Huang et al. (2019a)
			↑Antibiofilm property	
Multifunctional coatings with single material	Flavonoid quercitrin	Wet chemistry	↑Biocompatibility, cell adhesion, and osteocalcin production	Llopis-Grimalt et al. (2020)
			↓Adhesion and viability of *S. epidermidis*	
	*L. casei*	Culturing	↑Osteogenic differentiation	Tan et al. (2020)
			↑Antimicrobial effect	
	TiBP/AMPA	Binding by titanium-binding domain	↓Adverse host inflammatory immune response	Wisdom et al. (2020)
	TiBP/GL13K		↓Bacterial colonization and biofilm formation	
	HBPL	Silane coupling	Creating an alkaline microenvironment	Yang et al. (2021)
			↑Osseointegration	
			↑Damaging DNA of bacterial	
	FP	Cycloaddition	↑Vascularization and osseointegration	Chen et al. (2021a)
		Silane coupling	↑Antimicrobial effect	

#### Roughening/Porous Surface Loaded with Antibacterial Substance

The roughening surface facilitates the attachment of osteoblasts; therefore, the combination of antibacterial agents with porous surface is another general strategy to construct a dual-functional coating. As mentioned previously, we introduced the controlled release antibacterial coating. Some researchers doped osteogenic material into the controlled release antibacterial coating to achieve the multifunction surface. Shen et al. deposited magnesium/zinc on the surface of porous titanium and coordinated with 2,5-dihydroxyterephthalic acid (DHTA) to form a hybrid magnesium/zinc-metal organic framework (Mg/Zn-MOF74) coating (Shen et al., 2019). The dissolution of MOF74 coating was accelerated in an acidic environment. The gradual release of DHTA and Zn^2+^ formed an alkaline microenvironment around the implant to kill bacteria and promote osteoblast proliferation. Mg^2+^ improves osteoblast activity and anti-inflammatory gene expression. *In vivo* results showed that the coating had high antibacterial and anti-inflammatory properties at the initial stage of implantation, and greatly improved the new bone formation around the implant. Gao et al. fabricated a titanium dioxide nanospike coating on the titanium surface and immobilized cationic polypeptides on the surface by coordination (Gao et al., 2020). The results showed that this coating was able to rapidly kill pathogenic bacteria, inhibit biofilm formation for up to 2 weeks, and promote the formation of HA.

3D printing was introduced previously as a method to construct a given morphology on implant that promotes osseointegration. Embedding antibacterial ions into 3D-printed coatings is one of the methods to construct multi-functional coatings. Hengel et al. closely embedded silver and zinc nanoparticles into a 3D-printed porous titanium oxide layer. The porous structure could promote the adhesion and the released Zn^2+^ and Ag^+^, which would do favor to promote osteogenesis and achieve an antibacterial effect, respectively (van Hengel et al., 2020a). In addition, this synergistic mechanism apparently reduced the toxicity of Ag to host cells.

To achieve better bone-promoting properties, researchers combined other bone-promoting substances with antibacterial agents on the modified surface. Antibacterial agents that can be directly combined with these bone-promoting surfaces can be utilized to achieve multifunctional coatings. He et al. coated polydopamine, cationic antimicrobial peptide LL-37, and phospholipids on a MAO modified titanium surface, which showed good cytocompatibility to mesenchymal stem cells and osteoblasts (He et al., 2018). LL-37 killed bacteria by blocking the expression of bacterial related genes and enhancing immune response under the controlled release of phospholipids. Others hydrothermally grew ZnO nanorod arrays on titanium and modified them by autopolymerization of dopamine and covalent immobilization of RGDC peptides (Li et al., 2017). The results showed that ZnO nanorod arrays could kill bacteria by producing ROS to destroy the cell membrane, and Zn^2+^ that penetrate the cell membrane could inhibit their metabolism. What is more, Zn^2+^ bound with dopamine could promote cytocompatibility and minimize possible cytotoxicity *via* an antioxidant effect to scavenge ROS.

#### Osteogenic Antibacterial Polymer Coatings

A direct approach to fabricate dual-functional orthopedic implants is to co-immobilize antibacterial agents and bone-promoting substances on Ti implants. The most common strategy is to load osseointegration coating and then graft antibacterial agents on the modified surface. For instance, dimethylaminododecyl methacrylate (DMADDM) is a new antibacterial agent whose amino group and long alkyl chain can destroy the bacterial membrane, thus exerting a strong inhibitory effect to bacteria and fungi, including drug-resistant strains (Wang et al., 2017b; Cheng et al., 2017; Rego et al., 2017). To endow a HA modified surface with antibacterial property, DMADDM was introduced with the aid of polydopamine (PDA) (Zhou et al., 2020). DMADDM was gradually released in the first 4 weeks after implantation, exhibiting strong inhibition to the adhesion and proliferation of pathogenic bacteria. After 4 weeks, the samples induced osteogenic differentiation attributed to the bone-like HA. With the aid of an alternative approach, antimicrobial agents and osteogenic materials can be combined and loaded on Ti. Sung et al. functionalized titanium implants with poly(glycidyl methacrylate) (PGED) polymer brushes, and covalently coupled quaternized polyethyleneimine QPEI, an efficient cationic antibacterial agent rich in amino groups, and alendronate, which has a high affinity for bone minerals, to the polymer brushes (Sun et al., 2019). The coating did inhibit bacterial infection in the early stage and enhanced osseointegration in the later stage. Zhang et al. prepared Ca- and Si-based ceramic (CS) nanorod coatings on Ti using MAO technique, and loaded AMP onto CS coatings with the aid of fluorous-cured collagen scaffolds (Zhang et al., 2021). The release of Ca^2+^ and Si^2+^ enhanced osseointegration and the collagen scaffold loaded with AMP had a good antimicrobial effect while promoting cell adhesion.

It is worth noting that some researchers have achieved antibacterial and osseointegration by modulating the microenvironment around the implant. Tao et al. prepared a multifunctional hybrid coating through depositing the zeolitic imidazolate frameworks-67 (ZIF-67) and an osteogenic growth peptide (OGP) on nanotubes (Tao et al., 2021). Under the acidic environment, the coating gradually decomposed, releasing cobalt ions and forming an alkaline microenvironment to kill bacteria. At the same time, OGP inhibited the inflammatory response and promoted differentiation of MSCs, facilitating osseointegration during the late implantation phase.

#### Photoantibacterial and Osteogenic Coatings

Photosterilization includes photodynamic therapy and photothermal therapy mentioned earlier, which can effectively eradicate bacteria in the biofilm. The application of photosensitizer and osteogenic material on the surface of titanium can promote osseointegration after sterilization. Typical types of materials have been explored for multifunction surfaces, including TiO_2_. To the best of our knowledge, TiO_2_ has great photocatalytic properties. After surface treatment, TiO_2_ can not only be used as a photosensitizer but also promote osseointegration. However, TiO_2_ can generate ROS to eradicate bacteria under ultraviolet light irradiation, but it cannot be triggered by near-infrared light (Zhang et al., 2020). Han et al. chose photosensitizer MoS_2_ with broader spectral responses to modify the surface of TiO_2_ and used MAO and hydrothermal treatment to construct the composite collagen/polydopamine/MoS_2_-TiO_2_ (CPM-TiO_2_) coating on the surface of titanium (Han et al., 2021). Under the combined action of photodynamic and photothermal therapy, *S. aureus* in biofilm could be quickly eradicated both *in vivo* and *in vitro*. The collagen in the coating was shown to promote the adhesion and proliferation of osteoblasts. Zhang et al. prepared a titanium dioxide nano-shovel/quercetin/l-arginine coating and doped ytterbium (Yb) and erbium (Er) on the TiO_2_ nano-shovel array (Zhang et al., 2022). Under near-infrared II light irradiation, Yb and Er promoted the production of ROS, which could kill bacteria. Meanwhile, ROS catalyzed the release of nitric oxide (NO) free radicals from l-arginine, promoting angiogenesis and osseointegration. The nano-shovel structure and quercetin coupled to the surface via organosilanes promoted osteogenic differentiation of BMSCs while NO promoted angiogenesis and osseointegration. In addition, the complex of generated electrons and holes by TiO_2_ reduces its photocatalytic properties and limits the antibacterial effect (Anandan et al., 2013). Graphdiyne enhances the catalytic effects of metals and possesses osteoinductive potential (Gu et al., 2014). Based on this, the researchers synthesized TiO_2_/GDY nanofibers by electrostatic force (Wang et al., 2020). The combination of the two increased the production of photocatalytic ROS and prolonged the antibacterial effect, inhibiting the formation of methicillin-resistant *S. aureus* biofilms, while also promoting bone tissue regeneration. Red phosphorus (RP) can also be used for photoantibacterial coatings, which have efficient photothermal properties and biodegradability, and can exist for a long time in human body with no toxicity (Latiff et al., 2015). Huang et al. used IR780 as photosensitizer preparing a RP/IR780/RGDC coating on titanium surface (Huang et al., 2019a). In the experiment, IR780 produced ROS under irradiation of 808 nm laser and improved the temperature sensitivity of bacterial biofilm; RP produced photothermal effect by near-infrared (808 nm) radiation of 50°C, which cooperated with ROS sterilization without causing tissue damage; the existence of RGDC promoted osteogenesis.

### Multifunctional Coatings with Single Material

It is worth noting that, in addition to combining antibacterial agents and osteotropic substances on titanium surface, some researchers have achieved multifunctional effects by using only one substance. Flavonoids from plants have been shown to have the ability to inhibit the formation of biofilm and bacterial toxicity, and to enhance the efficacy of antibiotics by blocking the efflux pump to reverse the antibiotic resistance of bacteria (Górniak et al., 2019). Among them, the flavonoid quercitrin not only had antibacterial properties but also promoted bone and anti-fibrosis (Córdoba et al., 2015; Gomez-Florit et al., 2016). The researchers prepared quercitrin coating on the surface of porous titanium alloy, and it turned out that the quercitrin coating was able to prevent bacterial adhesion, inhibit bacterial activity, and improve the efficiency of surface osteogenesis (Llopis-Grimalt et al., 2020). Another novel method is to culture *Lactobacillus casei* on the surface of a heat-treated Ti to form an inactivated probiotic modified coating (Tan et al., 2020). This *L. casei* biofilm showed an excellent 99.98% antibacterial effectiveness against methicillin-resistant *S. aureus*. Furthermore, the polysaccharides in the biofilm could promote osteogenic differentiation through stimulating macrophages to secrete osteogenic factors.

Synthetic multifunctional peptides can be used as a single material for multifunctional coatings. Antimicrobial sequences are combined with bone-promoting sequences to form multifunctional peptides for immobilization on titanium surfaces to achieve multifunctional coatings (Zhang et al., 2018b; Liu et al., 2018; Wisdom et al., 2020). The RGD peptide is derived from extracellular matrix proteins, promoting mammalian cell adhesion as an integrin ligand (Atefyekta et al., 2019). Based on this, RGD was linked to the antimicrobial sequence from human β-defensin-3 as a multifunctional chimeric peptide (Zhang et al., 2018b). This chimeric peptide effectively prevented the formation of biofilms by inhibiting bacterial gene expression and induced osteoblast differentiation and mineral deposition. Some studies reported that antimicrobial peptides were covalently immobilized onto titanium surfaces, but direct conjugation of these peptides abolishes their antimicrobial activity (Chouirfa et al., 2019). Wisdom et al. developed high-affinity inorganic binding peptides (TiBP) containing a Ti-binding domain to recognize Ti surfaces (Wisdom et al., 2020). Then, they combined RGD and antibacterial peptides with TiBP, and proved that this biofunctional peptide has antibacterial and bone-promoting properties with good stability and durability. Another method of surface immobilization is silanization of the Ti surface, immobilizing organic molecules by covalent stabilization (Kucharíková et al., 2016). Yang et al. immobilized hyperbranched poly-l-lysine (HBPL) polymers in Ti *via* silane coupling agents (Yang et al., 2021). The poly-l-lysine was able to interfere with the integrity of bacterial membranes and produce ROS to damage DNA of bacterial, exhibiting efficient sterilization properties *in vivo* and *in vitro*. Furthermore, the degradation of the coating *in vivo* created an alkaline microenvironment around the implant, which promotes osseointegration compared with the control group. Chen et al. synthesized a fusion peptide (FP) containing the antimicrobial sequence and the angiogenic sequence by a cycloaddition and introduced an alkyl group on the Ti surface to immobilize FP (Chen et al., 2021a). *In vivo* tests proved that the fusion peptide was able to kill 99.63% of *S. aureus*, as well as promote vascularization and osseointegration.

## Discussion

Aseptic loosening and infection are still challenges in fabricating coatings for orthopedic and orthodontic implants, so we introduce the current strategies combating the aseptic loosening and infection of titanium-based implants in this review. Antibacterial metal ions combined with porous structure to achieve antibacterial properties and promote osseointegration are most widely used. However, burst release, cytotoxicity, short half-life, and limited stability are ongoing problems of metal ionbased strategies. These problems are addressed by coatings with controlled release properties and synergistic effects between the substances. It is worth mentioning that smart release coatings have become research hotspots recently but most of them do not exhibit sufficient capacity to promote osteogenesis. In the future, dual-function smart release coatings may become the focus of research. On the one hand, the coating can release antibacterial agents to kill bacteria in a slightly acidic environment suitable for bacteria; on the other hand, it can release osteogenic factors in a slightly alkaline environment that is conducive to osteogenesis. In addition, strategies to regulate the microenvironment around the implant also have to be mentioned, including the regulation to tissue and the local inflammatory system. Proper inflammation milieu will do facilitative effect to osseointegration, but excessive inflammation will cause serious tissue damage. Therefore, the search for an appropriate inflammatory microenvironment by modulating the polarization of macrophages is a new method to promote osteogenesis. Multifunctional coatings on titanium surfaces are formed based on biomolecules with good cytocompatibility; coatings designed by these strategies promote bone formation and provide antibacterial effects at the same time. Four main strategies are currently followed: the direct binding of antibacterial and bone-promoting substances to the surface of titanium, integrating antibacterial substances into roughening/porous surface modified coatings, photoantibacterial and osteogenic coatings, and multifunctional coatings with single material. Among them, multifunctional peptides have better bone-forming properties and avoid resistance formation, which are the shortcomings of traditional antibacterial agents. However, they still face challenges in terms of stability and long-term performance. New substances or surface modification methods with antibacterial and bone-promoting properties need to be explored, and will become the focus of research to simplify surgery, reduce costs, and provide more safety for the patient.
